# CD82 is a marker to isolate β cell precursors from human iPS cells and plays a role for the maturation of β cells

**DOI:** 10.1038/s41598-021-88978-y

**Published:** 2021-05-05

**Authors:** Ami Watanabe, Anna Tanaka, Chizuko Koga, Masahito Matsumoto, Yasushi Okazaki, Tatsuya Kin, Atsushi Miyajima

**Affiliations:** 1 Institute for Quantitative Biosciences, University of Tokyo, 1-1-1 Yayoi, Bunkyo-ku, Tokyo, 113-0032 Japan; 2 Graduate School of Medical and Dental Sciences, Department of Biofunction Research, Institute of Biomaterials and Bioenginnering, Tokyo Medical University and Dental University, 2-3-10 Kanda-Surugadai, Chiyoda-ku, Tokyo, 101-0062 Japan; 3 Diagnostics and Therapeutics of Intractable Diseases, Graduate School of Medicine, Intractable Disease Research Center, Juntendo University, 2-1-2 Hongo, Bunkyo-ku, Tokyo, 113-8421 Japan; 4 Clinical Islet Laboratory, University of Alberta Hospital, 210 College Plaza, 8215-112 St, Edmonton, AB T6G2C8 Canada; 5 Present Address: Gene Techno Science Co.,Ltd, Kita 21-jo Nishi 11-chome Kita-ku, Sapporo, 001-0021 Japan

**Keywords:** Induced pluripotent stem cells, Stem-cell differentiation, Endocrine system and metabolic diseases

## Abstract

Generation of pancreatic β cells from pluripotent stem cells is a key technology to develop cell therapy for insulin-dependent diabetes and considerable efforts have been made to produce β cells. However, due to multiple and lengthy differentiation steps, production of β cells is often unstable. It is also desirable to eliminate undifferentiated cells to avoid potential risks of tumorigenesis. To isolate β cell precursors from late stage pancreatic endocrine progenitor (EP) cells derived from iPS cells, we have identified CD82, a member of the tetraspanin family. CD82^+^ cells at the EP stage differentiated into endocrine cells more efficiently than CD82^−^ EP stage cells. We also show that CD82^+^ cells in human islets secreted insulin more efficiently than CD82^−^ cells. Furthermore, knockdown of CD82 expression by siRNA or inhibition of CD82 by monoclonal antibodies in NGN3^+^ cells suppressed the function of β cells with glucose-stimulated insulin secretion, suggesting that CD82 plays a role in maturation of EP cells to β cells.

## Introduction

Pancreatic islets consist of α, β, δ, and Pancreatic polypeptide (PP) endocrine cells and β cells are responsible for the glucose stimulated insulin (INS) secretion (GSIS). Autoimmune destruction of β cells causes type1 diabetes and the patients are treated by insulin administration on a daily basis. However, some patients with type 1 diabetes develop hypoglycemia unawareness, a life-threatening condition that is not easily treatable with insulin injection. For such patients, transplantation of pancreas or pancreatic islets is an effective treatment option. Pancreatic islets isolated from deceased donors are transplanted into the liver via the portal vein, which is less invasive than pancreatic transplantation. However, the limited availability of islets is a major obstacle to widespread islet transplantation. To obtain a large quantity of islets or β cells, pluripotent stem cells (PSCs), such as ES cells and iPS cells, have been considered as a promising cell source. Based on studies of signaling molecules involved in pancreatic development using mouse models and human fetal tissues, various protocols to generate islets or β cells from ES/iPS cells have been successfully developed^1–6^.

In general, differentiation in vitro is induced through multiple steps, i.e. definitive endoderm (DE), primitive gut tube (PG), pancreatic progenitors (PP), endocrine progenitors (EP) and maturation to β cells. Each step requires 2–10 days of incubation with a different set of costly cytokines, chemicals, and media. Because of multi-step processes and it is difficult to achieve 100% differentiation efficiency^7^, the final cell products are likely to include unnecessary cells such as undifferentiated cells that can result in adverse effects such as tumorigenesis upon transplantation. While the differentiation protocols of PSCs into islet cells have been established, a recent report using single cell analysis showed that there is a significant difference in gene expression profiles between human adult islets and islets derived from PSC^8^, It is thus possible that there are still rooms for refinement. As the maturation process of EP cells to fully functional β cells with GSIS is still not fully understood, in this paper, we have focused on EP cells derived from human iPS cells.

During the development of the mouse pancreas, all endocrine cells are derived from endocrine progenitor cells (EP) that highly express NEUROG3 (NGN3)^9–11^. Therefore, characterization of NGN3^+^ EP cells and studies on the maturation to endocrine cells in vitro will help improve the protocol to derive mature β cells from iPS cells. To characterize EP cells, it would be beneficial to find cell surface proteins that can be used to isolate late EP cells. In this study, we isolated the cells highly expressing NGN3 from hiPS-EP cells efficiently differentiated into pancreatic islet cells, particularly β cells. Islets derived from CD82^+^ cells showed GSIS in vitro. Furthermore, CD82 is expressed not only in late EP cells but also in adult islets, and CD82^+^ cells in adult pancreatic islets exhibited the ability to secrete more INS than CD82^−^ cells. Moreover, suppression of CD82 during the maturation process in vitro reduced GSIS. The results in this paper indicate that CD82 is a marker for late EP cells that can differentiate to mature β cells. We also provide evidence that CD82 plays an important role in β cell function.

## Results

### Isolation and characterization of cells highly expressing NGN3

**Figure 1:**
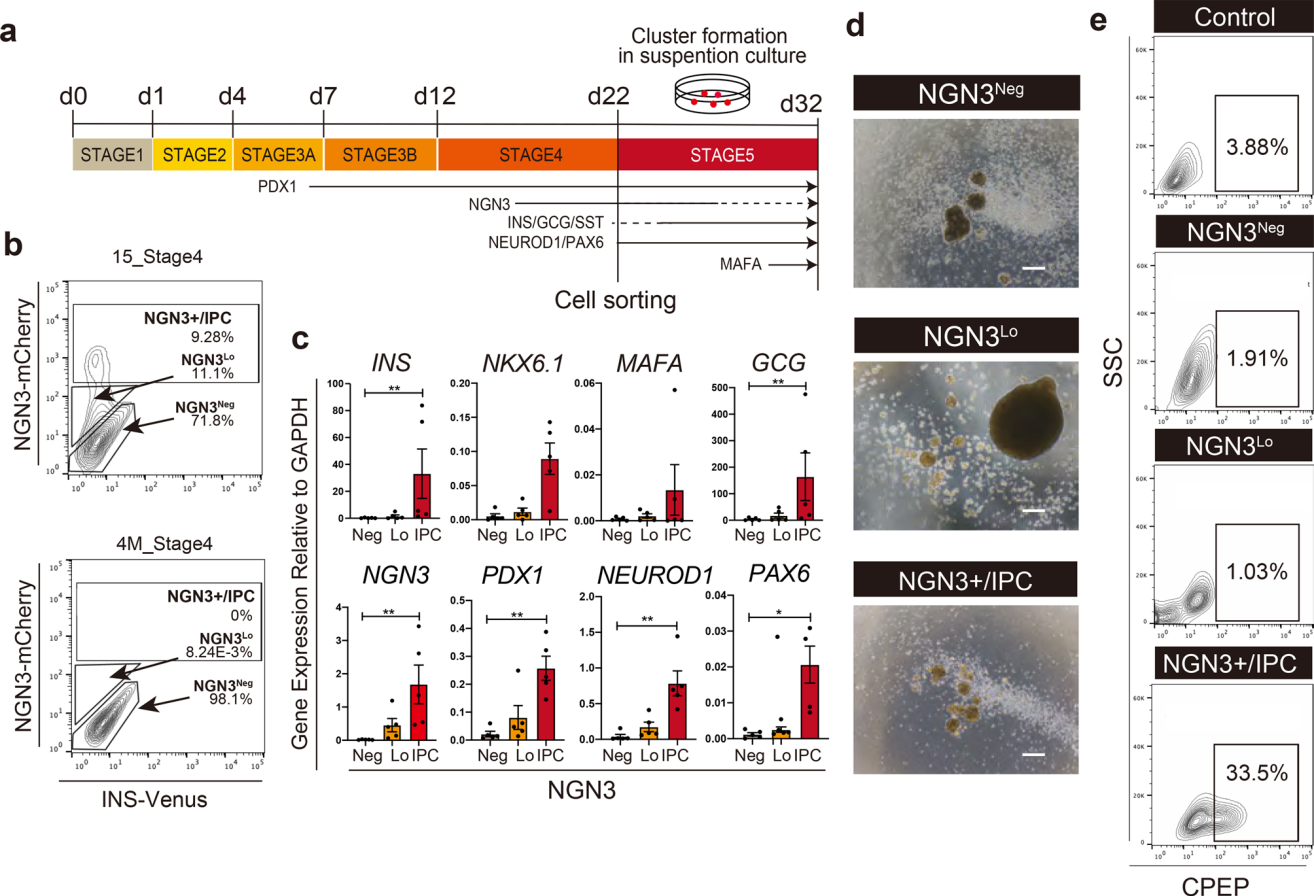
NGN3+/IPC cells contain pancreatic endocrine progenitor cells. (**a**) Differentiation scheme of pancreatic endocrine cells from hiPSCs. This figure was drawn by the authors. The software was powerpoint 2016.16.18. (**b**) Flow cytometric analysis of the EP stage cells derived from reporter iPS cells by expression of NGN3-mCherry and INS-Venus. Control shown is the EP stage cells derived from control iPS cells without reporter (TKDN-4M). (**c**) Gene expression analysis of the sorted NGN3^Neg^, NGN3^Lo^, and NGN3 + /IPC cell fractions. Expression levels are normalized by GAPDH. N = 5 independent biological replicates. The data shown are mean ± SEM. *P < 0.05, **P < 0.005, ***P < 0.0005. No mark is added for the data that are not significantly different. (**d**) Bright-field images of d32 NGN3^Neg^, NGN3^Lo^ and NGN3 + /IPC cells. (1 × 10^6^ cells/well, 6-well plate) Scale bar, 100 μm. (**e**) Flow cytometric analysis of clusters derived from NGN3+/IPC, NGN3^Lo^ and NGN3^Neg^ cells by staining with anti-CPEP antibody or isotype control.

In order to isolate EP cells from human iPS cells, we utilized a double knock-in human iPS reporter cell line, in which mCherry and Venus genes are inserted at the NGN3 and INS locus, respectively^12^. The reporter iPS cells were differentiated into the EP stage by our multi-step differentiation protocol (Fig. 1a). After 22 days of differentiation, EP stage cells were analyzed by flow cytometry to detect mCherry and Venus expression. NGN3 highly expressing cells (NGN3+/immediate progeny cells, NGN3+/IPC) that include some INS co-expressing cells and NGN3 low expressing cells (NGN3^Lo^) were 9.28% and 11.1% of EP stage cells, respectively (Fig. 1b). To confirm the specificity of mCherry signal, expression of genes related to pancreatic differentiation in sorted cells was examined by qRT-PCR. NGN3+/IPC cells expressed a higher level of the pancreas marker PDX1 and EP-endocrine markers, INS, GCG, NEUROD1, compared to NGN3 negative (NGN3^Neg^) and NGN3^Lo^ cells (Fig. 1c). Western blot analysis revealed that mcherry^+^ cells expressed higher level of the NGN3 protein compared with NGN3^LO^ cells (Fig. S1a). These results suggest that EP cells are enriched in the NGN3+/IPC fraction^13^.

Next, NGN3+/IPC and NGN3^Lo^ cells were induced to differentiate into pancreatic endocrine cells by our protocol based on the previous reports^1,2,14^. To induce maturation of the isolated endocrine precursor cells into endocrine cells, especially β cells, we developed a suspension culture (Fig. 1a, Stage5). NGN3+/IPC, NGN3^Lo^, NGN3^Neg^ cells were cultured for 10 days in shaking culture using a 6-well culture plate, resulting in the formation of cell clusters of NGN3+/IPC, NGN3^Lo^ and NGN3^Neg^ cells (Fig. 1d). Flow cytometry was performed to determine the proportion of β cells in those cell clusters (Fig. 1e). In the NGN3+/IPC clusters, c-peptide (CPEP), a short peptide in proinsulin positive β cells were 33.5%, whereas they were 1.03% in the NGN3^Lo^ clusters and 1.91% in the NGN3^Neg^. These results indicate that NGN3+/IPC cells in EP cells are able to differentiate to insulin-expressing β cells efficiently.

### Identification of a new cell surface marker of pancreatic late EP cells

**Figure 2:**
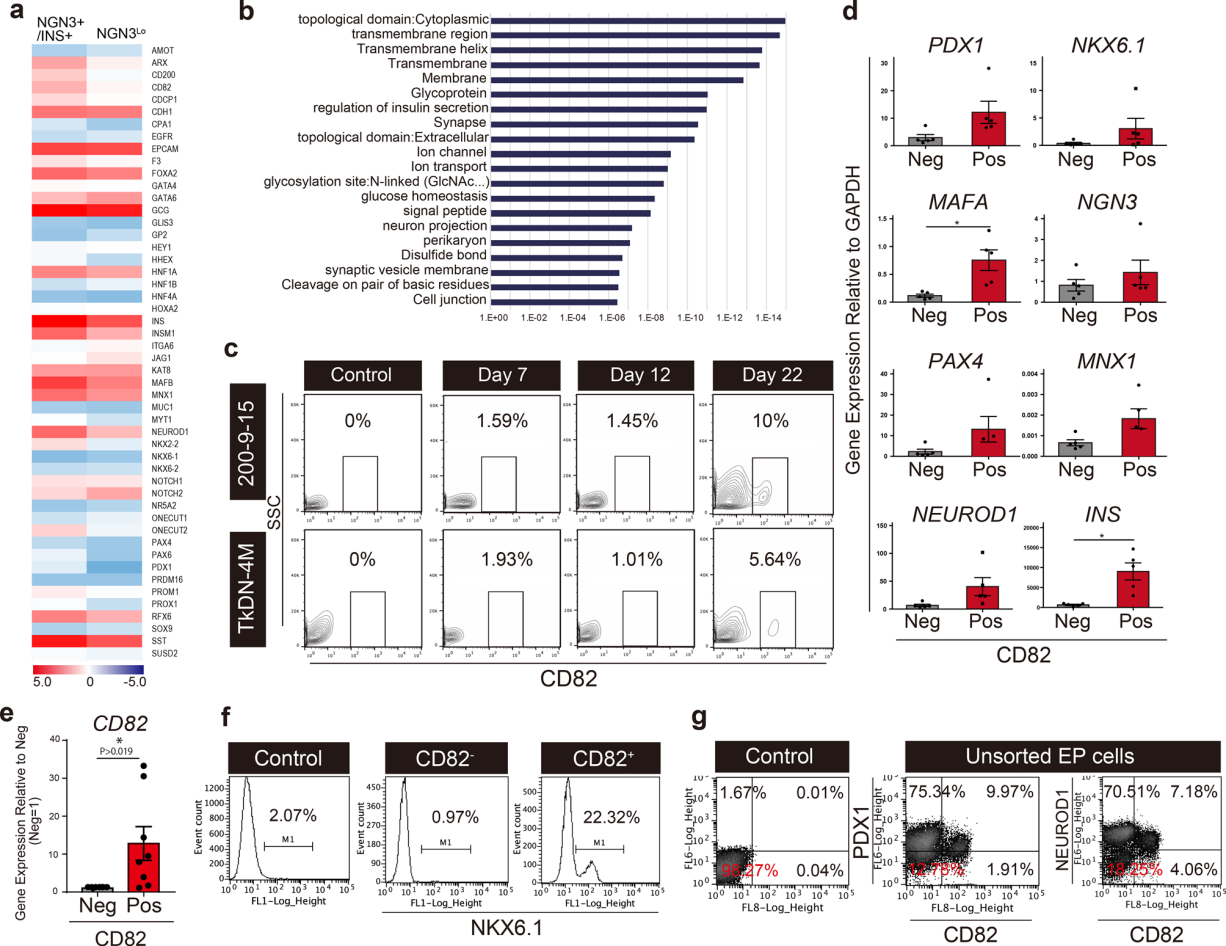
Characterization of CD82 expressing cells in hiPS-pancreatic progenitor cells. (**a**) Heat map of expression of the pancreatic progenitor related genes in the NGN3^+^INS^+^ (left) and NGN3^Lo^ (right). The color scale indicates the normalized expression value. (**b**) GO analysis showing enrichment of GO term in the NGN3^+^INS^+^ cells. Representative GO categories are shown and plotted against log (*P*-value). (**c**) Flow cytometric analysis of CD82-expressing cells in day 7, day 12, and day 22 cells. Results using two iPS cell lines are shown. Isotype control of d22 cells is shown). (**d**) Gene expression analysis of pancreatic genes in the CD82^−^ and CD82^+^ cells sorted from the pancreatic progenitor stage cells. Expression levels are normalized by GAPDH. N = 5 independent biological replicates. The data are shown as mean ± SEM. *P < 0.05. No mark is added for the data that are not significantly different. (**e**) CD82 expression analysis of pancreatic genes in the CD82^−^ and CD82^+^ cells sorted from the pancreatic progenitor stage cells. Expression levels are normalized by GAPDH. Data shown are the relative expression fold change CD82^−^ cells = 1. N = 8 independent biological replicates. The data are shown as mean ± SEM. *P < 0.05. (**f**) Gene expression analysis of pancreatic genes in the CD82^−^ and CD82^+^ cells sorted from the pancreatic progenitor stage cells. Expression levels are normalized by GAPDH. N = 5 independent biological replicates. The data are shown as mean ± SEM. *P < 0.05. No mark is added for the data that are not significantly different. Intracellular flow cytometric analysis of NKX6.1 in CD82^+^ and CD82^−^ sorted cells (d22). Isotype control of unsorted EP cells is shown. (**g**) Flow cytometry of CD82 vs PDX1 and NEUROD1 in EP stage cells. Isotype control of unsorted EP cells is shown.

In order to identify cell surface markers in pancreatic late endocrine progenitor cells, especially β cell progenitor, we performed microarray analysis of NGN3 and INS-Venus expressing cells fraction (NGN3^+^INS^+^) and NGN3^Lo^ and INS^Neg^ cells (Fig. S1b-d). The reason to add INS positive fraction for the analysis was that Ngn3 expression is known to decrease in β-progenitor cells and insulin gene expression is up-regulated along the maturation. Therefore, more mature candidate cells that become insulin-positive cells can express Ngn3. Therefore, we examined the population that include INS-positive and Ngn3-positive cells. NGN3^+^INS^+^ cells can differentiate into β cells efficiently compared with NGN3^Lo^ cells (Fig. S1e). Figure 2a shows a heat map of the expression of genes that are known to be expressed during pancreatic development. These results revealed that marker genes of pancreatic development were expressed at higher levels in NGN3^+^INS^+^ cells than NGN3^Lo^ cells (Fig. 2a). In particular, expression of mature endocrine cell markers, such as INS, MNX1, NEUROD1, GCG, SST, tends to be high in NGN3^+^INS^+^ cells. Markers of pancreatic endoderm (CD142) and pancreatic endocrine progenitor (CD200) were highly expressed in NGN3^+^INS^+^ cells compared with NGN3^Lo^. By contrast, expression of pancreatic progenitor (GP2) and endocrine progenitor (SUSD2) markers was low^13,15^. Next, we analyzed 768 genes that were expressed more than fivefold in NGN3^+^INS^+^ cells compared to NGN3^Lo^ cells. The NGN3^+^INS^+^ cell population was significantly enriched for GO-term of the signal peptide, membrane protein-related, glucose homeostasis-related genes (Fig. 2b). To select a cell surface marker highly expressed in NGN3^+^INS^+^ cells, 131 genes were filtered with the GO term “membrane” and “signal”. Among them, we selected CD82 because it is a membrane protein that has commercially available antibodies useful for cell sorting. CD82 is a member of the tetraspanin protein family and is known as a suppressor of various cancer metastasis^16–19^. However, its role in pancreatic development is unknown.

To characterize the CD82^+^ cell population in each differentiation step, flow cytometry was performed. CD82 was hardly detected at day 7 and 12, but it was expressed at day 22 (Fig. 2c). Similar results were obtained in the two different iPS cell lines (Fig. 2c). Gene expression analysis using qRT-PCR revealed that the markers related to pancreatic endocrine cells, MAFA and INS, were expressed significantly higher in the CD82^+^ cells compared to CD82^−^ cells (Fig. 2d). Also, In CD82^+^ cells, CD82 expression in CD82^+^ cells was significantly higher compared with CD82^-^ cells (Fig. 2e).

Flow cytometry analysis confirmed the protein expression of NKX6.1 in CD82^+^ cells (Fig. 2f). FACS analysis of the CD82^+^ and CD82^-^ cells revealed that Nkx6.1 positive endocrine progenitor cells were concentrated in CD82^+^ fraction cells (Fig. 2f). Interestingly, in unsorted EP cells, the majority of CD82^+^ cells were PDX1^+^ and NEUROD1^+^, but not all PDX1^+^ cells and NEUROD1^+^ cells expressed CD82 (Fig. 2g). This result suggests that CD82^+^ cells are a subset of pancreatic endocrine progenitor cells and immature β cells.

### Differentiation of CD82^+^ cells into pancreatic endocrine clusters

**Figure 3:**
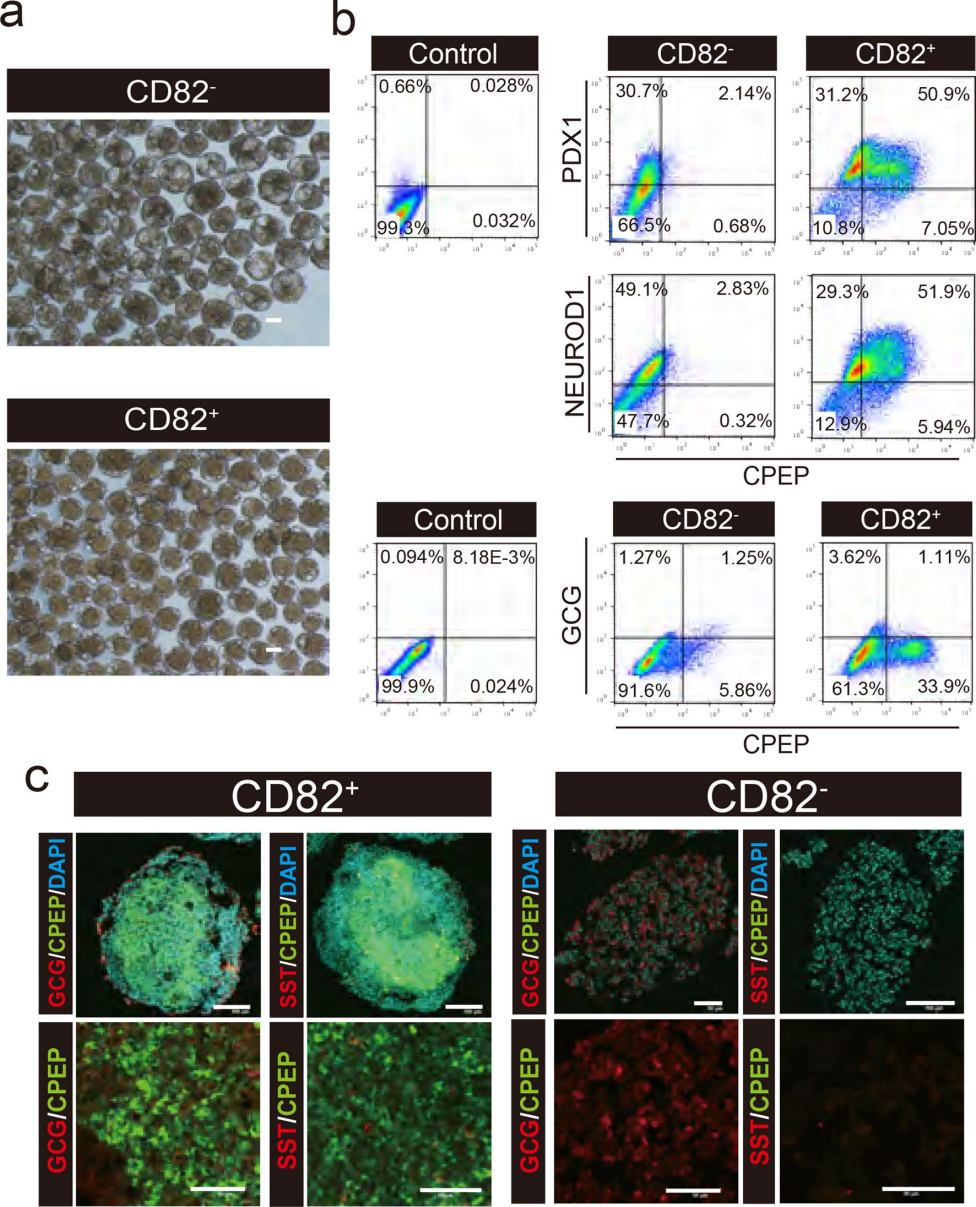
Differentiation of CD82^-^ and CD82^+^ cells into pancreatic endocrine cell clusters. (**a**) Bright-field images of cell clusters derived from CD82^−^ and CD82^+^ cells. Scale bar, 100 μm. (**b**) Intracellular flow cytometric analysis of CD82^−^ and CD82^+^ cell clusters co-stained with anti-PDX1/anti-CPEP, anti-NEUROD1/anti-CPEP, and anti-GCG/anti-CPEP antibodies. Isotype control of unsorted EP cells is shown. (**c**) Immunofluorescence staining of the clusters derived from CD82^−^ and CD82^+^ cells. CPEP (green), GCG/SST (red). Top panel: low-power field. Bottom panel: high-power field. Scale bars, 100 μm (Top) and 50 μm (bottom).

To test whether CD82^+^ cells can differentiate into mature endocrine cells, CD82^+^ and CD82^−^ cells were induced to differentiate to pancreatic endocrine cells. CD82^+^ and CD82^−^ cells were cultured in a 6-well plate by shaking. Under this condition, CD82^+^ and CD82^−^ cells formed cell clusters within 24 h. Like NGN3 + /IPC and NGN3^+^INS^+^ cells, CD82^+^ cell formed uniform clusters with smooth surface (Fig. 3a). Flow cytometry analysis revealed that CPEP/PDX1 double-positive β cells and NEUROD1/CPEP double-positive β cells were 50.9% and 51.9% of CD82^+^ cells, respectively. In contrast, CPEP/PDX1 double-positive and NEUROD1/CPEP double-positive cells in CD82^-^ clusters were 2.14% and 2.83%, respectively.

The glucagon (GCG) positive α cells were 4.73% of CD82^+^ cells and 2.52% of CD82^-^ cells. These data indicate that CD82^+^ cells differentiated into β cells more efficiently than CD82^−^ cells. CD82^−^ clusters contained PDX1^+^ cells but very few β cells. Previous studies reported that pancreatic differentiation from PSCs and pancreatic development in vivo generates polyhormonal cells co-expressing GCG and INS^20,21^. In our case, the cells co-expressing INS and GCG were 1.11% of CD82^+^ cells and 1.25% of CD82^-^ cells (Fig. 3b). These results showed that a few polyhormonal cells were present in both clusters of CD82^+^ and CD82^-^ cells. We further examined the localization of endocrine cells in the clusters by immunostaining and found that the CD82^+^ clusters consisted of monohormonal β, α, δ cells, whereas there was almost no β cells in the CD82^-^ cell clusters (Fig. 3c). These results indicate that CD82 is a useful marker to purify for late EP cells with a potential to differentiate into mature endocrine cells, especially β cells.

### CD82 expression defines a distinct subset of pancreatic progenitors

**Figure 4:**
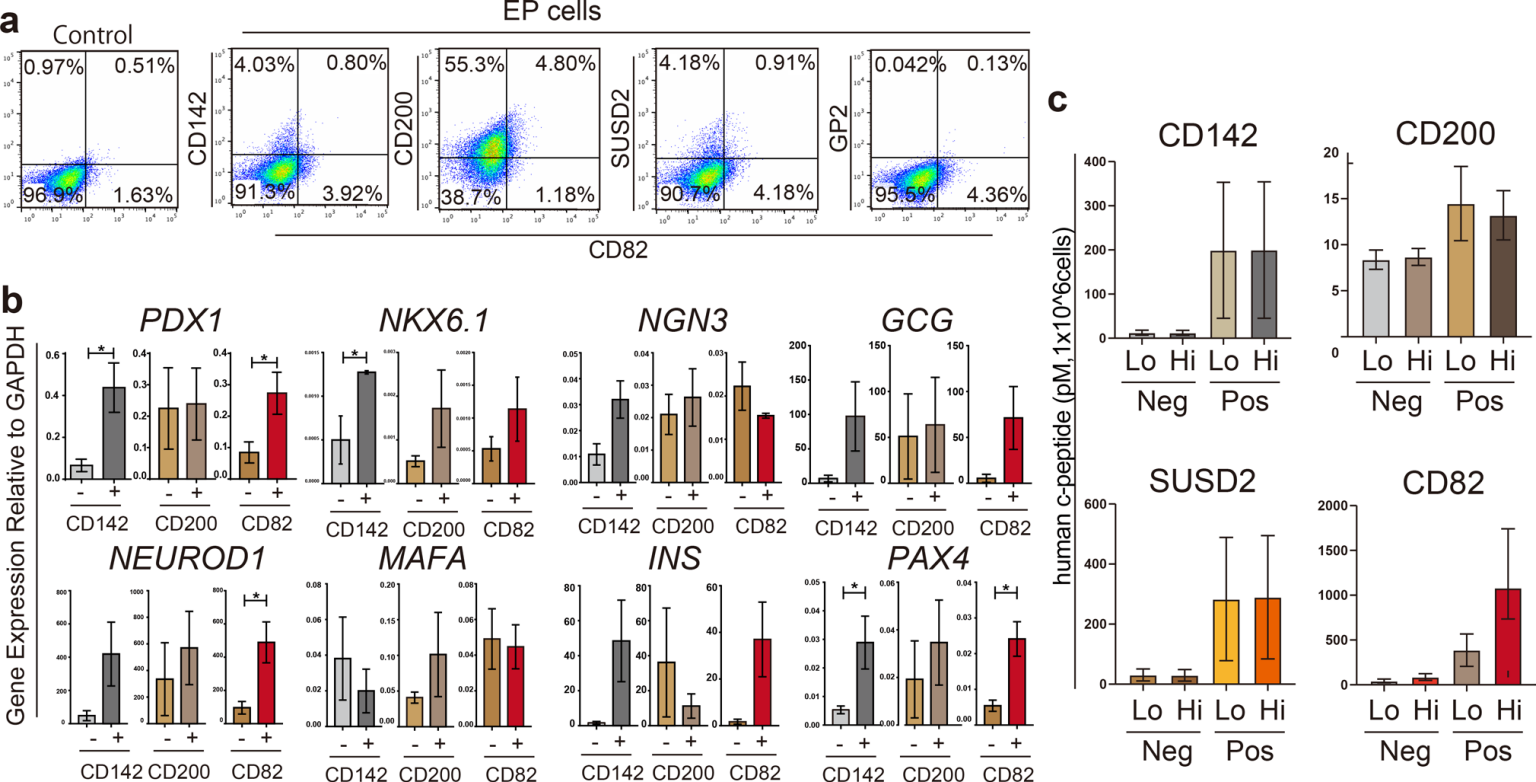
A Comparison of CD82 with other pancreatic progenitor markers. (**a**) Flow cytometric analysis of EP cells stained with anti-CD82 and anti-CD142, anti-CD200, anti-SUSD2, anti-GP2 antibodies. Isotype control of unsorted EP cells is shown. (**b**) Gene expression analysis of cells sorted by expression of CD142, CD200, and CD82. N = 3 independent biological replicates. The data are shown as mean ± SEM. No mark is added for the data that are not significantly different. (**c**) ELISA analysis of human C-peptide in clusters derived from either positive or negative cells sorted by expression of CD142, CD200, SUSD2, and CD82. Lo; 2.8 mM glucose medium, Hi; 28 mM glucose medium (see experimental procedures). The data are shown as mean ± SEM (N = 3–4) (1 × 10^6^ cells per assays). *P < 0.05, No mark is added for the data that are not significantly different.

Previous studies reported immature pancreatic progenitor markers such as CD200, CD142, GP2, and SUSD2^13,15,22^. The cells expressing such a marker were shown to differentiate into mature pancreatic endocrine cells in vitro or in vivo. To investigate the relationship between CD82 and those that previously reported as progenitor markers, we compared CD82 expression with other markers in the EP cells by flow cytometry (Fig. 4a), revealing that CD82 was expressed in part of CD200 cells, but not expressed CD142 and SUSD2 expressing cells. These results suggest that CD82 may be expressed on the cells at a differentiation stage distinct from the cells with known markers.

The expression levels of the EP genes in CD82^+^ cells were comparable to those cells expressing either CD142 or CD200 (Fig. 4b). We next investigated the differentiation of sorted cells using different cell surface markers, CD82, CD142, CD200 and SUSD2, in the 96-well suspension culture system. In the maturation culture, cell clusters derived from all the sorted cells secreted C-peptide (Insulin). Interestingly, CD82^+^ cells secreted C-peptide at a much higher level compared to cell clusters from cells with the other markers (note the different vertical scale in Fig. 4c). These results indicate that CD82^+^ cells exhibit different characteristics from the previously reported cells with either CD200, CD142 or SUSD2.

### Expression of CD82 in mature pancreatic islets

**Figure 5:**
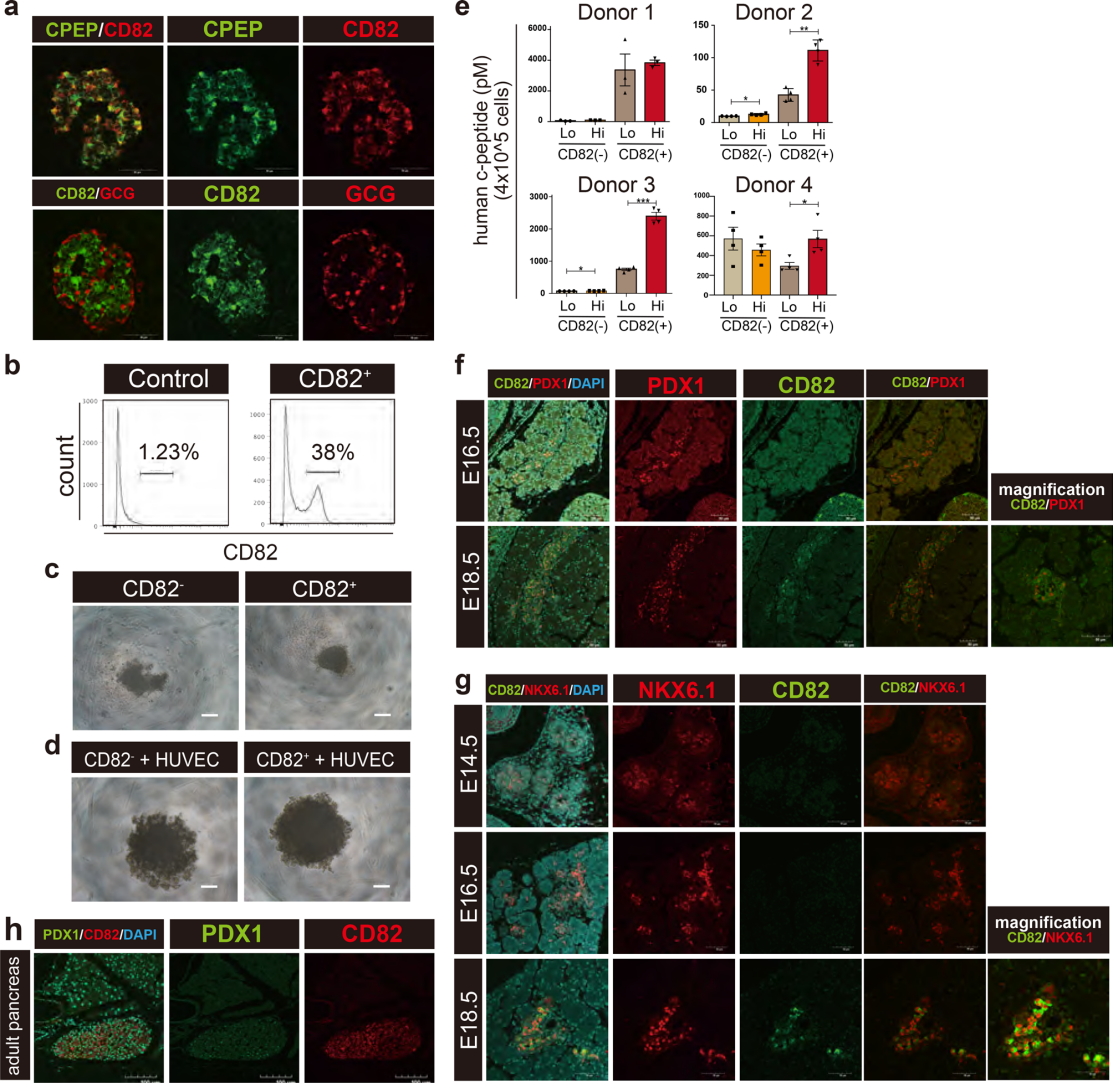
Expression of CD82 in adult human islets and mouse late fetal pancreas. (**a**) Immunostaining of human pancreas section. C-PEP (green), CD82 (Red) (Upper panel), and GCG (Red), CD82 (Green) and DAPI (Blue) (Lower panel). Scale bar 50 μm. (**b**) Flow cytometric analysis of human islet cells stained with the anti-CD82 antibody. Isotype control of dissociated islet cells is shown. (**c**) Bright-field image of clusters derived from CD82 positive or negative pancreatic islet cells at 24 h after re-aggregation. Scale bar 100 μm. (**d**) Bright-field image of clusters derived from CD82 positive or negative human pancreatic islet cells co-cultured with HUVEC, 5 days after re-aggregation. Scale bar 100 μm. (**e**) ELISA analysis of GSIS from clusters derived human islets (normalized 1 × 10^6^ cells per assay). Lo; 2.8 mM glucose reaction solution, Hi; 28 mM glucose (see experimental procedures). Representative results of islets from 4 donors are shown. N = 3–4 technical replicates. Data are presented as mean ± SEM. *P < 0.05, **P < 0.005, ***P < 0.0005. No mark is added for the data that are not significantly different. (**f**) Immunostaining of fetal mouse pancreas section (E16.5, E18.5). CD82 (green), PDX1 (red), nucleus (DAPI). Scale bar 50 μm. (**g**) Immunostaining of fetal mouse pancreatic sections at E14.5, E 16.5, and E 18.5. CD82 (green), NKX6.1 (red), nucleus (DAPI). Scale bar 50 μm. (**h**) Immunostaining of adult mouse pancreatic sections. CD82 (red), PDX1 (green), nucleus (DAPI, Blue). Scale bar 100 μm.

CD82 is expressed in the normal adult pancreas islets, but its function in pancreatic endocrine cells has remained unknown^23^. We investigated the CD82 expression in adult human islets by immunostaining and revealed that CD82^+^ cells were detected in all C-peptide^+^ β cells and a few GCG^+^ cells (Fig. 5a). CD82 was not localized to the nucleus and appeared to be distributed in the cytoplasm and near the cell membrane. Next, we fractionated human islets cells based on CD82 expression (Fig. 5b). CD82^+^ cells represented 38% of the total islet cells.

Next, we investigated the C-peptide secretion from human pancreatic CD82^+^ and CD82^−^ cells. However, the dissociated single β cells failed to secrete insulin^24^. Therefore, we examined whether the isolated cells could be reaggregated in the 96-well floating culture condition. Interestingly, we found that only CD82^+^ cells formed compact cell clusters (Fig. 5c), indicating a difference in aggregation ability between human pancreatic CD82^+^ and CD82^−^ cells. It was recently reported that HUVEC and MSC induced aggregation of dissociated islet cells^25,26^. Based on these findings, we co-cultured isolated islet cells with HUVEC at the 1 1 ratio, resulting in the formation of clusters of CD82^+^ and CD82^−^ cells in 24 h (Fig. 5d). We assessed C-peptide release from these clusters of 4 donors and found that CD82^+^ clusters tend to secrete more C-peptide in the high glucose concentration than in the low glucose concentration (Fig. 5e). In contrast, CD82^−^ clusters tend to no difference with low glucose and high glucose stimulation. These results suggest that CD82 may be a marker that can identify the β cells with GSIS in the adult pancreas.

In order to know the timing of CD82 expression during development, we investigated CD82 expression in mouse pancreatic development. Immunostaining of fetal mouse sections revealed that CD82 was not expressed in the developing pancreatic tissue before E16.5. CD82 appeared in the vicinity of PDX1^+^ cells in the E18.5 pancreas and was co-expressed with NKX6.1, a transcription factor of endocrine progenitors (Fig. 5f,g). In adult mouse islets, CD82 was expressed in islets area (Fig. 5h). These results suggest that CD82 is an immature β-cell marker that is expressed from the late EP stage in the developmental stage.

### Role of CD82 in functional maturation of β cells

**Figure 6:**
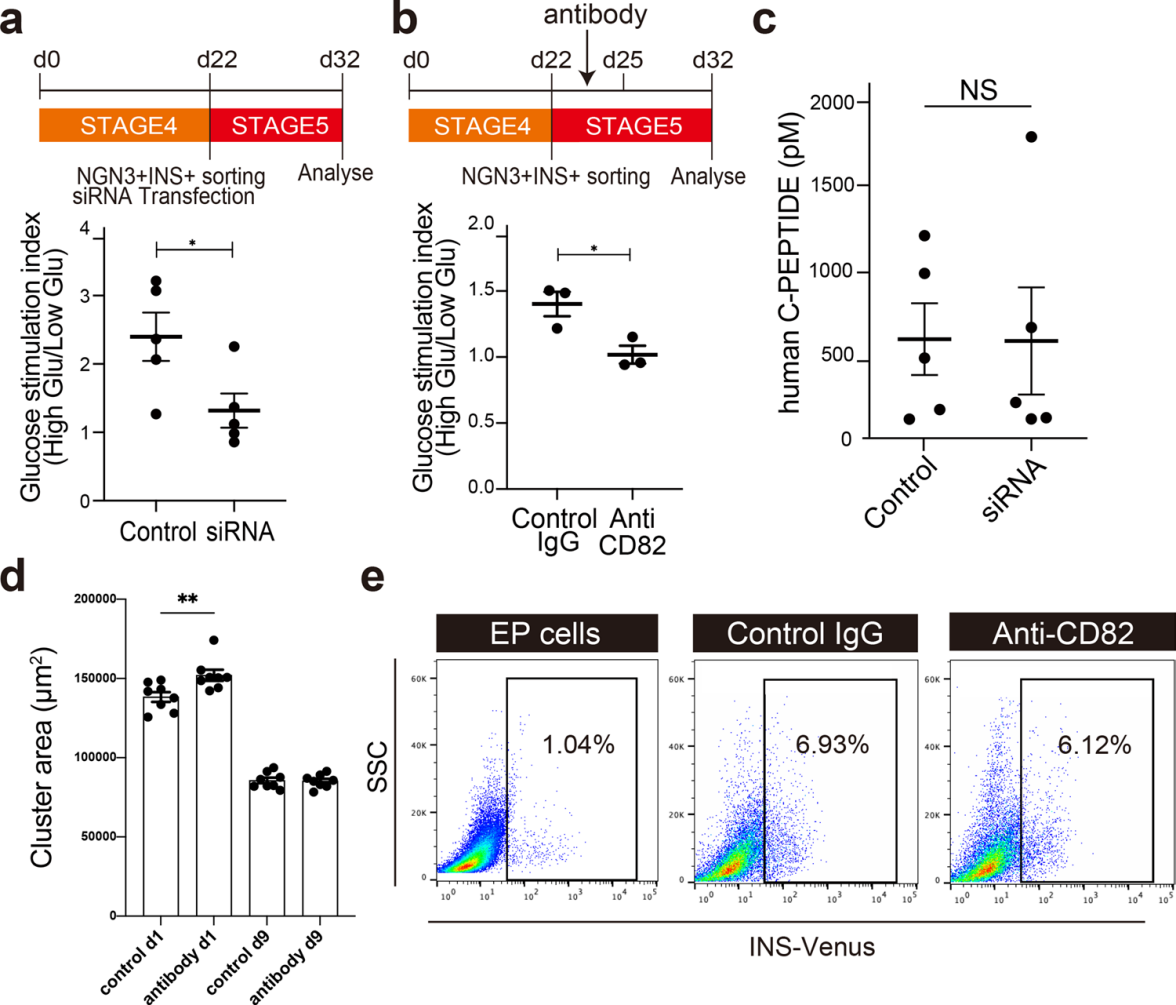
Role of CD82 in functional maturation of β cells. (**a**) Glucose-stimulated insulin (C-peptide) secretion index of in clusters of CD82 knockdown cells (1 × 10^6^ cells per assays). Data shown are the relative secretion fold change (Low glucose = 1), representative 5 biological replicates. Data are presented as mean ± SEM. *P < 0.05. No mark is added for the data that are not significantly different. (**b**) GSIS index of anti-CD82 antibody treated cells (1 × 10^6^ cells per assays). Data shown are the relative secretion fold change (Low glucose = 1), representative 3 biological replicates. Data are presented as mean ± SEM. *P < 0.05. No mark is added for the data that are not significantly different. (**c**) ELISA analysis of high glucose-stimulated C-peptide secretion (28 mM) from CD82 knockdown clusters and control clusters (1 × 10^6^ cells per assays). Related data to (**a**). N = 5 independent biological replicates. The data are presented as mean ± SEM. (**d**) The sizes of EP cell clusters. Cluster sizes were estimated by brightfield images of CD82 inhibited clusters or control by using Photoshop. 15 clusters from 3 differentiation batches were measured. Representative data were shown. The data are presented as mean ± SEM. No mark is added for the data that are not significantly different. (**e**) Flow cytometry analysis of INS-Venus in the CD82 knockdown clusters. EP cells; non-treated EP stage cells.

Because CD82 was found to be expressed from the late EP stage to mature β cells, we addressed whether CD82 might play a role for the differentiation/maturation of EP cells. As shown in Fig. 1, NGN3^+^INS^+^ cells were purified from a differentiation culture dish at d22 and NGN3^+^INS^+^ cells were transfected with CD82 siRNA to knockdown CD82 expression. The cells were then matured for 10 days in suspension culture and C-peptide secretion was examined. Knocking down CD82 expression in NGN3^+^INS^+^ cells reduced glucose responsiveness (Fig. 6a, Fig S2a, and Fig. S3a). Consistent with this result, inhibition of CD82 using an inhibitory antibody in EP cells suppressed glucose responsiveness (Fig. 6b, FigS3b). To investigate the effect of CD82 inhibition on C-peptide secretion from mature β cells, mature endocrine cell clusters at d32 were incubated with inhibitory antibodies for 3 days. However, CD82 inhibition did not affect GSIS (Fig. S2b). These data suggest that inhibition of CD82 in the maturation stage blocks the acquisition of GSIS.

We further investigated the effect of CD82 in detail. The inhibition of CD82 did not affect C-peptide secretion and the size of cell clusters after maturation (Fig. 6c,d, Fig. S3c) and flow cytometry analysis indicated no difference in the fractions of β cells in the clusters between the control group and the antibody-treated group (Fig. 6e). Furthermore, qRT-PCR analysis showed that CD82 suppression did not significantly affect RNA expression of INS and GCG and β-cell maturation markers such as ERRγ, FLTP, and VAMP2 (Fig. S2c)^27–29^. These results suggest that suppression of CD82 during the β cell maturation inhibits GSIS. However, in mature β cells, CD82 suppression did not inhibit GSIS. These results suggested that CD82 may play a role for β cells to acquire glucose responsive insulin secretion.

## Discussion

Islet transplantation is an effective treatment for severe type 1 diabetes, however donor shortage is a major problem. Because of the unlimited proliferation potential of PSCs, pancreatic islets or β cells from PSCs are a promising cell source for cell therapy. However, as the differentiation of PSCs to β cells in vitro is achieved by lengthy and multi-step processes, the efficiency of differentiation to islets can be variable. Furthermore, the final cell product includes not only pancreatic endocrine cells but also other types of cells such as undifferentiated cells, which can result in unexpected adverse event when transplanted. For the clinical use of iPS-derived β cells, those contaminated cells need to be eliminated to avoid potential risks of tumorigenesis. Therefore, it should be beneficial to develop a means to purify pancreatic endocrine progenitor cells that can differentiate to islets for cell therapy.

In this study, we have focused on the NGN3^+^ and INS^+^ late endocrine progenitors that give rise to β cells. To isolate and characterize late EP cells, we utilized a double knock-in human iPS reporter cell line, in which mCherry and Venus genes were inserted at the NGN3 and INS locus, respectively^12^. NGN3^+^ cells were further fractionated into NGN3 + /IPC and NGN3^Lo^ cells based on the mCherry expression level. Sorted NGN3 + /IPC cells formed tight clusters and differentiated to multiple endocrine cells in the suspension 3D culture. Several markers of pancreatic development, especially β cell markers, were highly expressed in NGN3 + /IPC cells. We then performed a microarray analysis to find genes specifically expressed in NGN3^+^INS^+^ cells. Among those genes, we selected CD82 for further studies, because it is a membrane protein with commercially available antibodies useful for cell sorting. The CD82 gene (also known as KAI1, TSPAN27, C33, ST6, IA4) encodes a 267-amino acid protein containing four transmembrane domains, belonging to the tetraspanin family. CD82 is widely expressed in normal tissues and moderately expressed at the mRNA level in the pancreas. We found that CD82 was expressed in differentiated cells after the EP stage. In mouse development, CD82^+^ cells were found in the pancreatic region after E18.5 (Fig. 5f,g), indicating that CD82 is expressed in late endocrine precursor cells in mice, slightly later than E14.5–16.5, when NGN3 expression peaks. Furthermore, in E18.5 mouse fetus and adult, CD82 is co-expressed with Nkx6.1 and PDX1, suggesting that CD82 is likely to be expressed in for late endocrine progenitor cells committed to β cells.

Several cell surface molecules, such as CD142, CD200, GP2, and SUSD2, have been reported as markers of pancreatic progenitor cell/pancreatic endocrine progenitor cells. In comparison with these markers, CD82^+^ cells appeared in the late EP stage and differentiated more efficiently into insulin-secreting cells. CD82^+^ cells rarely expressed these marker genes, suggesting that CD82 is expressed at a stage later than those characterized by previously reported markers. We also demonstrated that CD82 is expressed in human pancreatic islets and that β cells are enriched in CD82^+^ cells. Moreover, CD82^+^ β cells in adult islets tended to exhibit better GSIS and insulin secretion compared to the CD82^-^ cells. Thus, our results demonstrate that CD82 is a cell surface marker for separating β-cell precursors from pluripotent stem cell-derived EP cells and also purifying effective endocrine cells from adult islets.

In mouse development, CD82 expression was restricted to β cells after E18.5, suggesting that CD82 may be involved in functional maturation process of insulin secretory capacity. Surprisingly, during mouse development, CD82 expression was found in most of the E18.5 Nkx6.1-positive β progenitors, strongly suggesting a link between CD82 and pancreas developmental. Because it has been noted that in vitro differentiation does not fully mimic in vivo development, CD82 expression in the in vitro endocrine progenitor cell population may be limited.

An unexpected and interesting finding in this study is that suppression of CD82 expression or function in EP reduced GSIS in β cells. Tetraspanin family proteins are known to regulate cell morphogenesis, proliferation, invasion, fusion, adhesion, and signal transduction^30–32^. Since CD82 is down-regulated in advanced cancers, its role as a suppressor of tumor metastasis has been studied in detail^33–35^, whereas its role in pancreatic development has remained unknown. We show that CD82 inhibition during β cell maturation did not affect insulin (C-peptide) secretion, but did decrease GSIS. Also, suppression of CD82 did not affect the expression of known markers of glucose-stimulated insulin release of β cells, such as ERRδ and VAMP2. ERRδ has been reported as an important driver of the mitochondrial metabolic gene network of mature β cells, and forced expression of ERRδ allowed iPS-β cells to acquire the ability to secrete insulin^27^. Therefore, CD82 may play a role for the maturation of the insulin secretory pathway other than mitochondrial metabolism. It still remains unknown what role CD82 plays in β cells to acquire GSIS. Since CD82 is involved in cell migration and cell–cell interactions, it may function in a similar mechanism during development. However, inhibition of CD82 suppressed GSIS without affecting the proliferation, cluster formation, and gross insulin production. Thus, CD82 may play a role in glucose response.

The role of CD82 in the differentiation process from endocrine progenitor cells to beta cells remains unclear. It is also thought that CD82 expression is downstream of Ngn3, but this point still remains to be established. Furthermore, not all CD82 + cells differentiated into beta cells, suggesting that the CD82 + cell population may be heterogeneous. To clarify these important questions, lineage-tracing experiments may be useful. Such experiments will provide a clear view on the maturation process of pancreatic endocrine progenitors.

Clinical application of PSC-islets requires large scale production. As the differentiation from PSC to islets is a lengthy process consisting of multi-steps that require expensive cytokines, chemicals and media, the cost to produce PSC-islets is extremely high and hence practical use of those cells needs substantial cost reduction. Proliferation potential of iPS cells declines along differentiation and EP cells do not proliferate. Therefore, it would be beneficial if EP cells can be propagated without losing their ability to differentiate to islets. Recently, we have shown that NGN3^+^ EP cells can be expanded by expression of SV40LT and removal of SV40LT after the expansion allowed the cells to differentiate to mature β cells^36^. We are currently investigating whether sorted CD82^+^ EP cells can be expanded. Isolation and expansion of CD82 + late EP cells from PSC will be able to substantially reduce the production cost of islets and eliminate unnecessary cells to avoid unexpected adverse event in transplantation.

## Materials and methods

### Cell culture and differentiation

Human iPS cell lines, 200-9-15^12^ and TkDN4-M^37^ were used for all experiments. Undifferentiated iPS cells were cultured in Matrigel-coated dishes at 37 °C with 5% CO2. iPS cells were maintained in mTESR1 and differentiation to EP cells was started within DMEM/F12 medium with 25% KSR, 0.1 mM non-essential amino acid (GIBCO), 100 × PSG(GIBCO), 55 μM β-mercaptoethanol (GIBCO), 5 ng/ml FGF10 (Day 0). Differentiation was induced by changing medium as follows: Day 1 (Stage1); RPMI1640 medium (FUJIFILM) containing 100 ng/ml activin A (PEPROTECH) and 10 μM CHIR99021 (FUJIFILM). Days 2–4 (Stage2); RPMI1640 medium containing 10% B27 supplement (GIBCO) and 100 ng/ml activin A. Days 5–7 (Stage3A); DMEM (high-glucose, FUJIFILM) containing 50 μM FGF10 (PEPROTECH) (only on the day 5), SANT1 (SIGMA), 0.25 μM EC23 (Reinnervate), 6 μM SB431542, 1 μM Dorsomorphin (FUJIFILM) and 10% B27 supplement. Days 8–14 (Stage3B); 5 μM Repsox (ABCAM) was added to the Stage3A medium. Day 15–25 (Stage4); DMEM medium containing SANT1 (SIGMA), 1 μM Dorsomorphin (FUJIFILM), 5 μM Repsox, 50 ng/ml IGF-1 (FUJIFILM), 50 μM FGF10 (PEPROTECH, Day 15 only), 50 ng/ml Exendin 4 (PEPTIDE INSTITUTE), 10 μM DAPT (TOKYO KASEI) ,10 μM Folskolin (FUJIFILM) and 10% B27 supplement. Day 26–36 (Stage5); DMEM/F12 medium (FUJIFILM) with Exendin-4 (50 ng/ml), 10% B27 supplement, 0.5 mM HEPES (GIBCO), 1xPSG (GIBCO), 2 μM nicotinamide (FUJIFILM), 55 μM β-mercaptotol (GIBCO), 50 ng/ml GLP1 (PEPTIDE INSTITUTE), 5 μM Repsox, 0.25 μM SANT1, 2 nM Caspase-3 Inhibitor Z-DEVD-FMK (R&D), 10 μM Folskolin and 50 ng/ml IGF-1.

EP cell and Purified Ngn3 and INS expressing cell were differentiated in Stage5 medium in 6 well flat-bottom cell culture plates placed on a on a rotary shaker (OPTIMA) with shaking at 85 rpm/min. in case of small-scale culture, cells were static cultured in 96 well low attach plate (MS-9096 M, SUMITOMO BAKELITE).

### Glucose-stimulated C-peptide secretion

First, cells of a prefixed number were collected by a cell sorter (differentiated cell clusters: 1 × 10^6^ cells, Human pancreatic islets: 4 × 10^5^ cells). The cells were clustered in a shaking culture and used for the experiment. Human pancreatic islets or differentiated cell clusters from iPS cells were washed twice with Krebs–ringer solution and then pre-incubated for 2 h in low glucose (2.8 mM). The cell clusters were washed twice with glucose-free Krebs–ringer solution, incubated in low glucose Krebs–ringer solution for 1 h, and supernatants were removed. The cells were then incubated in 1 ml of low glucose medium for 45 min, and supernatants were collected, followed by incubated in 1 ml of high glucose (28 mM) Krebs–ringer solution for 45 min and the supernatant was collected. In some cases, cells were again incubated in a low glucose Krebs–ringer solution for 45 min. Insulin in the supernatants were detected using Human Ultrasensitive C-peptide ELISA and Human C-peptide ELISA (MERCODIA).

### Human islets

The use of human pancreatic islets was approved by the Health Research Ethics Committee of the University of Alberta. The all patients or their family’s informed consent was obtained by Dr. T. Kin (Clinical Islet Laboratory, University of Alberta Hospital). Human pancreas donated to the University of Alberta were used to prepare pancreatic islets according to the protocol approved by the Health Research Ethics Committee of the University of Alberta. Isolation of CD82^+^ cells from islets was carried out at the University of Tokyo according to the guideline of the Life Science Research Ethics and Safety at the University of Tokyo. Upon arrival, islets were incubated for 10 min at 37 °C in 1 × TrypLE select (GIBCO), followed by dissociation of pancreatic islets by pipetting. Cells were isolated using a Moflo cell sorter. HUVEC was added to isolated pancreatic islets at a ratio of 1: 1 and placed in a low attach 96-well M plate (SUMITOMO BAKELITE) at 50,000 cells/well. Phenol red-free RPMI medium (SIGMA) supplemented with 10% FBS (GIBCO) and 1% PSG (GIBCO) was used as a medium. The next day cell clusters were collected for analysis.

### Immunohistochemistry

For immunohistochemistry, cell clusters were fixed with 4% PFA for 1 h at 4 °C, washed, dehydrated, embedded in paraffin and sectioned for histological analysis. Before staining, paraffin was removed with xylene and ethanol. For staining, slides were blocked with PBS containing 10% donkey serum for 1 h at room temperature and incubated with primary antibody at 4 °C overnight. After washing with PBS, the secondary antibody was incubated at 37 °C for 2 h. After washing with PBS, it was sealed with Fluoromount (DIAGNOSTICS BIOSYSTEMS) containing DAPI.

For whole-mount staining, cell clusters were fixed with 4% PFA for 1 h at room temperature. Prior to intracellular staining, the cell clusters were treated with 0.5% Triton solution for 1 h at room temperature. The clusters were then stained with a primary antibody containing 10% donkey serum-PBS. After washing twice with PBS, they were stained with the secondary antibody at 37 °C for 2 h. After washing with PBS, cells were treated with PBS containing × 1000 DAPI. Representative images were taken using an OLYMPUS FV3000 confocal microscope. The Antibodies used are shown in Table S1. For immunofluorescence analysis of tissue sections, commercially available human and mouse pancreas sections were used (human sections : BIOCHAIN INSTITUTE INC, mouse pancreas sections : GENOSTAFF).

### Flow cytometry and cell sorting

Differentiated cell clusters or pancreatic islets were dispersed in a single cell suspension by incubation in 10xTrypLE Select at 37 °C for 10 min. For cell sorting, cells were re-suspended in DMEM medium containing 10% FBS and 100 × PSG. The cells were then re-suspended in 10% FBS/DMEM with primary antibody and incubated at 4 °C for 10 min. After washing twice with medium, cells were incubated with secondary antibodies at 4 °C for 10 min. After washing twice, cells were analyzed by using a cell sorter Moflo (BECKMAN COLTER). We collected the cells to the required number. Data were analyzed by the FlowJo software. When acquiring data, we created an FSC/SSC plot and excluded debris. For intracellular staining, cells were prepared using a commercially available kit from BD (Cytofix / Cytoperm Kit). The Antibody list are shown in Table S1. Experiments were performed multiple times, and representative data are shown in the figure.

### RNA extraction and real-time qPCR

Total RNA was extracted by using the RNeasy micro kit (QIAGEN). cDNA was reverse transcribed using TAKARA PrimeScript II 1st strand cDNA Synthesis Kit. Real-time PCR was performed with each primer at 10 nM and 10 × SYBR Green I (LIFE TECHNOLOGIES). Data are presented as mean expression ± SEM. Relative gene expression was determined by GAPDH expression, a housekeeping gene. The primer sequences used are shown in Table S2.

### Gene expression analysis by microarray

Total RNA was isolated from NGN3 + /IPC and NGN3^Lo^ cells using Qiashredder and RNeasy Mini Kit (QIAGEN). Reverse transcription and amplification of total RNA were performed using the Low Input Quick Amp Labeling Kit (AGILENT). Hybridization was performed with SurePrint G3 Human Gene Expression v3 8 × 60 K Microarray kit and scanned after staining. Microarray analysis was performed by ONCOMICS Inc. Microarray data have been deposited at GSE142367. Microarray data were analyzed by functional annotation of DAVID (https://david.ncifcrf.gov/home.jsp). Enriched functions were defined by a q-value below 0.05. The bar graph shows the level of *p* value.

### Knockdown of CD82 using siRNA in differentiated hiPSCs

Dissociated NGN3+/IPC cells were transfected with 20 nM CD82 siRNA or BLOCK-iT Fluorescent Oligo (INVITROGEN) using Human Stem Cell Nucleofector Kit 1 (LONZA). Medium was changed every day after transfection. After 10 days, glucose-stimulated insulin (C-peptide) secretion assay and FACS analysis of the cells was performed. SiRNA was used Stealth RNAi siRNA (Assay ID:HSS105654, HSS105654,THERMO FISHER SCIENTIFIC).

### Inhibition of CD82 using antibodies

CD82 neutralizing antibody LS‑B15130, LS‑204296 and LS‑C742189 (LSBIO) or mouse IgG (BIOLEGEND) were added to NGN3^+^ cells at a concentration of 10–20 μg/ml, followed by3 days culture. The cells were then cultured in Stage5 medium for 7 days and subjected to GSIS analysis and measurement of cluster size.

### Western blot analysis

NGN3 + /IPC, NGN3^Lo^, and EP stage cells were dissolved in RIPA buffer (1 × 10^6^ cells/ml, NACARAI TESQUE). Cell lysate were loaded on the Bolt 4–12 Bis-Tris Plus gels (INVITROGEN) and transferred to PVDF membrane by iBlot2 Transfer Stacks (INVITROGEN). After that, membrane blocking for 1 h in 5% Dry milk/PBS. Then, membrane was incubated with primary antibody against human NGN3(SANTACRUZ, #sc-376607) and human GAPDH (CALBIOCHEM, CB1001). After washing the membrane, membrane was incubated with second antibody against anti-mouse IgG (KPL, 474-1806), and Signals were then prepared by ECM solution (immobilon, MILLIPORE). Chemiluminescent Signals were detected by the Chemidoc Touch Imaging system (BIO-RAD).

## Supplementary Information

Supplementary Figures.
