# Supplementary material for: CD82 is a marker to isolate β cell precursors from human iPS cells and plays a role for the maturation of β cells
Source: Sci Rep. 2021 May 5;11:9530. doi: 10.1038/s41598-021-88978-y (PMC8100138; doi:10.1038/s41598-021-88978-y)
Supplement: Supplementary file 1 — Supplementary Figures. [file 41598_2021_88978_MOESM1_ESM.pdf]

Supplementary Figures

Title

CD82 is a marker to isolate β cell precursors from human iPS cells and plays a role for the maturation of β cells.

Authors

Ami Watanabe[1]*, Anna Tanaka[1], Chizuko Koga[1], Masahito Matsumoto[2], Yasushi Okazaki[3], Tatsuya Kin[4], Atsushi Miyajima[1]*

[1]Institute for Quantitative Biosciences, University of Tokyo
1-1-1 Yayoi, Bunkyo-ku, Tokyo 113-0032 Japan

[2]Graduate School of Medical and Dental Sciences, Department of Biofunction Research, Institute of Biomaterials and Bioenginnering,
Tokyo Medical University and Dental University, Japan
2-3-10 Kanda-Surugadai, Chiyoda-ku, Tokyo 101-0062

[3]Diagnostics and Therapeutics of Intractable Diseases, Intractable Disease Research Center, Juntendo University, Graduate School of Medicine, Japan
2-1-2 Hongo, Bunkyo-ku, Tokyo 113-8421

[4]Clinical Islet Laboratory, University of Alberta Hospital
210 College Plaza, 8215-112 St, Edmonton, Alberta, Canada T6G2C8

Fig. S1

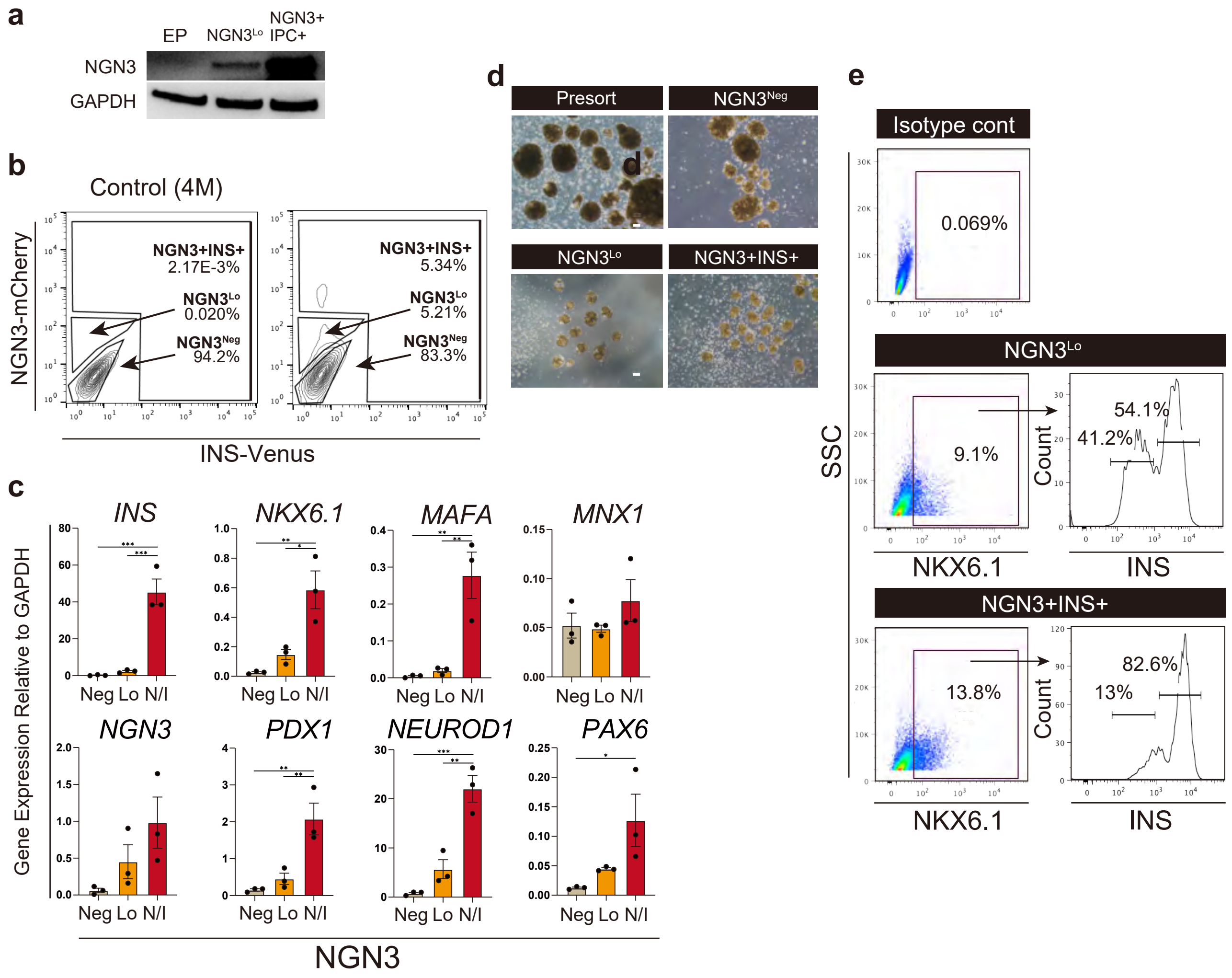

a
EP
NGN3Lo
NGN3+ IPC+
NGN3
GAPDH
b
Control (4M)
NGN3-mCherry
INS-Venus
NGN3+INS+ 2.17E-3%
NGN3Lo 0.020%
NGN3Neg 94.2%
NGN3+INS+ 5.34%
NGN3Lo 5.21%
NGN3Neg 83.3%
c
Gene Expression Relative to GAPDH
INS
NKX6.1
MAFA
MNX1
NGN3
PDX1
NEUROD1
PAX6
Neg Lo N/I
NGN3
d
Presort
NGN3Neg
NGN3Lo
NGN3+INS+
e
Isotype cont
0.069%
NGN3Lo
9.1%
54.1%
41.2%
NGN3+INS+
13.8%
82.6%
13%
SSC
Count
NKX6.1
INS

Fig. S1 Characterization of NGN3+INS cells. Related to Fig.1

A) NGN3 expression analysis in the NGN3+/IPC, NGN3Lo, and non-sorting EP stage cells.
B) Flow cytometric analysis of NGN3-mCherry and INS-Venus in the EP stage cells derived from reporter iPS cells.
C) Gene expression analysis of the sorted NGN3^{Neg} (Neg), NGN3^{Lo} (Lo), and NGN3+ INS (N/I) cell fractions. Expression levels are normalized by GAPDH. Data of 3 independent biological replicates are shown as mean ±SEM. *P<0.05, **p<0.005, ***p<0.0005. No mark is added for the data that are not significantly different.
D) Bright-field images of d32 NGN3^{Neg}, NGN3^{Lo} and NGN3+INS+ cells and presort cells. Scale bar, 100 μm.
E) Intracellular flow cytometric analysis of NKX6.1 and INS in clusters derived from NGN3 + INS and NGN3^{Lo} cells.

Fig.S2

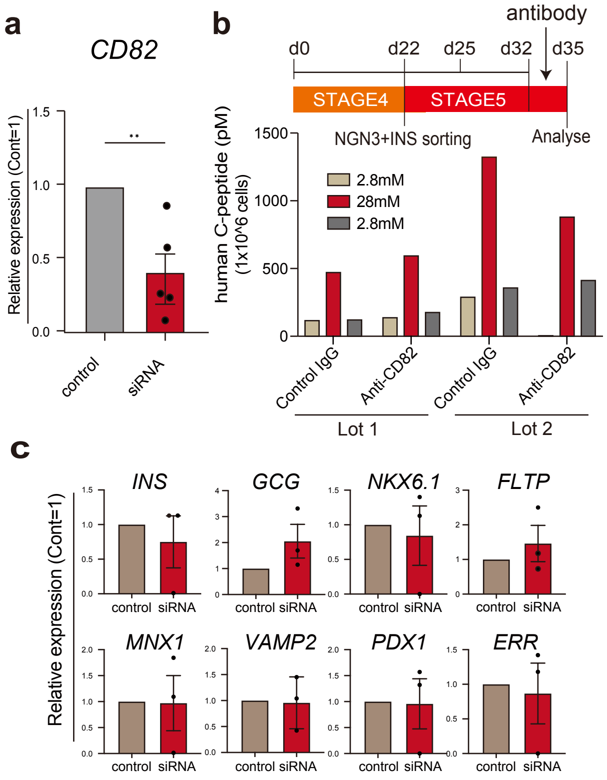
a
CD82
Relative expression (Cont=1)
1.5
1.0
0.5
0.0
**
control
siRNA
b
antibody
d0
d22
d25
d32
d35
STAGE4
STAGE5
NGN3+INS sorting
Analyse
human C-peptide (pM)
(1x10^6 cells)
1500
1000
500
0
2.8mM
28mM
2.8mM
Control IgG
Anti-CD82
Control IgG
Anti-CD82
Lot 1
Lot 2
c
Relative expression (Cont=1)
INS
GCG
NKX6.1
FLTP
MNX1
VAMP2
PDX1
ERR
control siRNA

Fig. S2 Analysis of CD82 knockdown cells. Related to Fig. 6

A) Expression of CD82 in CD82 knockdown NGN3+INS+ cells (qRT-PCR). Data of 5 independent biological replicates are shown as mean ± SEM. Related to Fig. 6A,C.
B) Secretion of C-peptide from clusters in the presence of low and high glucose concentration. Clusters were formed form cells treated with or without anti-CD82 antibody. Representative ELISA data obtained with two independent experiments (Lot1 and Lot2). Dissociated $1x10^6$ cells were used for each assay.
C) Gene expression analysis of the CD82 knockdown cells. Data of 3 independent biological replicates are shown as mean ±SEM. No mark is added for the data that are not significantly different.

Fig. S3

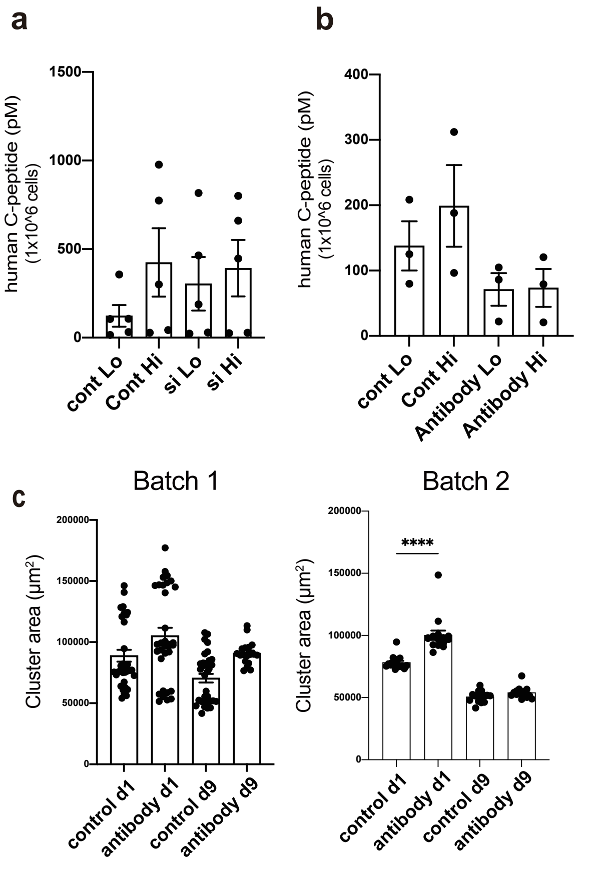
a
human C-peptide (pM)
(1x10^6 cells)
1500
1000
500
0
cont Lo
Cont Hi
si Lo
si Hi
b
400
300
200
100
0
cont Lo
Cont Hi
Antibody Lo
Antibody Hi
c
Batch 1
Cluster area (µm²)
200000
150000
100000
50000
0
control d1
antibody d1
control d9
antibody d9
Batch 2
****

Fig. S3 Role of CD82 in functional maturation of β cells. Related to Fig 6.

A) ELISA analysis of in clusters of CD82 knockdown cells ($1x10^6$ cells per assays). Related to Fig6a. Representative of 5 biological replicates. Data are presented as mean ± SEM. *P<0.05. No mark is added for the data that are not significantly different.

B) ELISA analysis of anti-CD82 antibody treated cells ($1x10^6$ cells per assays). Related to Fig. 6b. Representative of 3 biological replicates. Data are presented as mean ± SEM. *P<0.05.

C) The sizes of EP cell clusters. Related to Fig. 6c. Cluster sizes were estimated by brightfield images of CD82 inhibited clusters or control by using Photoshop. 15-22 clusters from 3 differentiation batches were measured. Data using two batches are shown. The data are presented as mean ± SEM.

Fig. S4

a

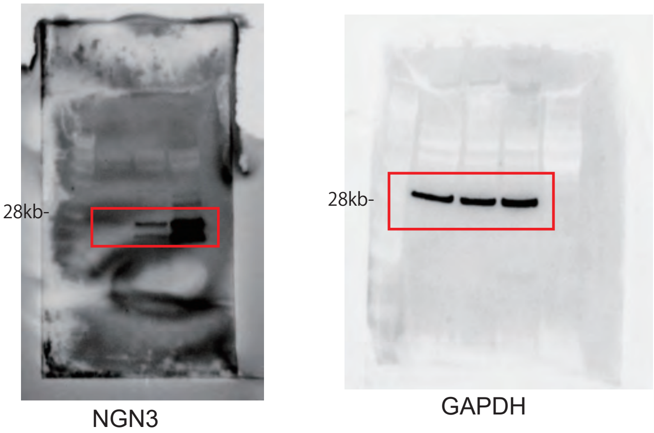

Fig. S4 Characterization of NGN3+INS cells. Related to Fig.S1a

A) Full length blot of NGN3 and GAPDH expression analysis in the NGN3+/IPC, NGN3Lo, and non-sorting EP stage cells.

TableS1 Antibodies used for flow cytometry and/or immunofluorescence imaging

antigen	species	source	#	conc.
Polyclonal Guinia Pig Anti-insulin	Polyclonal Guinia Pig	DAKO	A0564	x1000
Polyclonal Rabbit Anti-Glucagon	Polyclonal Rabbit	DAKO	A0565	x2000
Polyclonal Rabbit Anti-Somatostatin	Polyclonal Rabbit	DAKO	A0566	x1000
Anti-pro-insulin C-Peptide, Clone C-PEP-01, Mouse Monoclonal	monoclonal Mouse	Millipore	05-1109	x100
Anti-CD82 antibody	Rabbit polyclonal	abcam	ab66400	x50
Anti-PDX1 antibody	Guinea pig polyclonal to PDX1	abcam	ab47308	x100
Anti-hPDX-1	Polyclonal Goat IgG	R&D	AF2419	x50
Nkx6.1	monoclonal Mouse	DSHB	F55A12-s	x50
APC anti-human SUSD2 Antibody	Monoclonal mouse	biolegend	327408	x100
APC anti-human CD142 Antibody	Monoclonal mouse	biolegend	365206	x100
PE/Cy7 anti-human CD82 Antibody	Monoclonal mouse	biolegend	342110	x200
Alexa Fluor® 647 anti-human CD82 Antibody	Monoclonal mouse	biolegend	342108	x200
Purified anti-human CD82 Antibody	Monoclonal mouse	biolegend	342102	x200
Human CD200 APC-conjugated Antibody	Monoclonal mouse	R&D	FAB27241A	x100
GP2 Antibody (GP2/1712) APC	Monoclonal mouse	Novus	NBP2-54416APC	x100
Anti-NeuroD1 antibody	Monoclonal mouse	abcam	ab60704	x100
APC Mouse IgG2a, κ Isotype Ctrl Antibody		biolegend	400220	
FITC Mouse IgG2b, κ Isotype Ctrl Antibody		biolegend	401205	
PE/Cy7 Mouse IgG1, κ Isotype Ctrl Antibody		biolegend	400125	

TableS2 qPCR primers used for qPCR analysis.

gene symbol		sequence
INS	FW	GGGAGGCAGAGGACCTG
	RV	CCACAATGCCACGCTTCT
GCG	FW	GCTGCCAAGGAATTCATTGC
	RV	CTTCAACAATGGCGACCTCTTC
SST	FW	ACCCCAGACTCCGTCAGTTT
	RV	ACAGCAGCTCTGCCAAGAAG
NKX6.1	FW	CTGGCCTGTACCCCTCATCA
	RV	CTTCCCGTCTTTGTCCAACAA
GAPDH	FW	AGCCACATCGCTCAGACAC
	RV	GCCCAATACGACCAAATCC
NEUROD1	FW	TCCGGAGGCCCCAGG
	RV	CGCCCATCAGCCCACTC
PDX1	FW	AAGTCTACCAAAGCTCACGCG
	RV	GTAGGCGCCGCCTGC
MAFA	FW	GCCCTCCTTCGTTCTCTTCT
	RV	GGCTTCCTCCAAGGTTTTCT
NGN3	FW	GCTCATCGCTCTCTATTCTTTTGC
	RV	GGTTGAGGCGTCATCCTTTCT
MNX1	FW	CAGGAAGCGGAGAAACAGAA
	RV	GGGTCACTGTCCCTCAAGTC
PAX4	FW	CAGGAGGACCAGGGACTACC
	RV	GAGCCACTATGGGGAGTGAG
CD82	FW	GCTCATTCGAGACTACAACAGC
	RV	GTGACCTCAGGGCGATTCA
FLTP	FW	GCCAACGATCGTGGTC
	RV	CCCATGAAGGAACCCC
VAMP2	FW	CCAAGCTCAAGCGCAAAT
	RV	GGGATTTAAGTGCTGAAGTAAACTATG
ERR	FW	GCTAACACTGTCGCAG
	RV	CGAACAGCTGGAATCA
